# Glycogen Synthase Kinase-3β Inhibition with 9-ING-41 Attenuates the Progression of Pulmonary Fibrosis

**DOI:** 10.1038/s41598-019-55176-w

**Published:** 2019-12-12

**Authors:** Ann Jeffers, Wenyi Qin, Shuzi Owens, Kathleen B. Koenig, Satoshi Komatsu, Francis J. Giles, Daniel M. Schmitt, Steven Idell, Torry A. Tucker

**Affiliations:** 1 The Texas Lung Injury Institute, Tyler, TX USA; 2 Department of Cellular and Molecular Biology, The University of Texas Health Science Center at Tyler, Tyler, TX USA; 3 Actuate Therapeutics Inc, Fort Worth, TX USA

**Keywords:** Target validation, Growth factor signalling

## Abstract

Idiopathic pulmonary fibrosis (IPF) is a progressive interstitial lung disease with a median survival of 3 years after diagnosis. Although the etiology of IPF is unknown, it is characterized by extensive alveolar epithelial cell apoptosis and proliferation of myofibroblasts in the lungs. While the origins of these myofibroblast appear to be diverse, fibroblast differentiation contributes to expansion of myofibroblasts and to disease progression. We found that agents that contribute to neomatrix formation and remodeling in pulmonary fibrosis (PF); TGF-β, Factor Xa, thrombin, plasmin and uPA all induced fibroblast/myofibroblast differentiation. These same mediators enhanced GSK-3β activation via phosphorylation of tyrosine-216 (p-Y216). Inhibition of GSK-3β signaling with the novel inhibitor 9-ING-41 blocked the induction of myofibroblast markers; α-SMA and Col-1 and reduced morphological changes of myofibroblast differentiation. In *in vivo* studies, the progression of TGF-β and bleomycin mediated PF was significantly attenuated by 9-ING-41 administered at 7 and 14 days respectively after the establishment of injury. Specifically, 9-ING-41 treatment significantly improved lung function (compliance and lung volumes; p < 0.05) of TGF-β adenovirus treated mice compared to controls. Similar results were found in mice with bleomycin-induced PF. These studies clearly show that activation of the GSK-3β signaling pathway is critical for the induction of myofibroblast differentiation in lung fibroblasts *ex vivo* and pulmonary fibrosis *in vivo*. The results offer a strong premise supporting the continued investigation of the GSK-3β signaling pathway in the control of fibroblast-myofibroblast differentiation and fibrosing lung injury. These data provide a strong rationale for extension of clinical trials of 9-ING-41 to patients with IPF.

## Introduction

Idiopathic pulmonary fibrosis (IPF) is the most common form of interstitial lung disease. The overall incidence and prevalence of IPF in the United States is 14.0–42.7 and 16.3 per 100,000 persons, respectively^1–4^. The median survival in IPF ranges from 2.5 to 3.5 years after diagnosis with a median age at death of 65 years. The mean age of presentation at initial diagnosis is 63 years with familial cases presenting earlier at 59.4 years. Over 30,000 people die annually from IPF in the United States. While several treatments such as pirfenidone and nintedanib^2^ have been identified and are used clinically, they are not curative and are only known to slow the progression of this deadly disease. These considerations justify the search for new targets and novel pharmacologic interventions to more effectively treat IPF and reduce its mortality.

Chronic infiltration by and proliferation of myofibroblasts result in progressive PF. These cells express increased amounts of α-SMA and promote the accumulation of matrix proteins, including collagen, that contribute to fibrotic remodeling. This damage eventually culminates in destruction of alveolar architecture in IPF with formation of fibrotic foci and respiratory compromise with impaired gas exchange. Upon activation, lung fibroblasts can transition into myofibroblasts that are largely responsible for the increased collagen and matrix synthesis and deposition found in PF.

GSK-3 is a serine/threonine kinase that was first identified as a regulator of glycogen metabolism and insulin signaling operating through the regulation of glycogen synthase. GSK-3 has two isoforms, α and β^5–10^. GSK-3β functions overlap with those of GSK-3α but the converse is not true as GSK-3β knockout mice, unlike GSK-3α-deficient mice, are embryonically lethal^8^. Although GSK-3β is a constitutively active kinase, its activity is potentiated by phosphorylation of the tyrosine 216 residue (Y216)^11^. GSK-3β is also reported to regulate numerous transcription factors including NFκB, CREB, myocardin and myocardin-related transcription factors in different organs and systems^5,6,12–16^. Aberrant GSK-3β activity contributes to a wide range of pathological conditions including Alzheimer’s disease, diabetes mellitus, and carcinogenesis^6,17^. 9-ING-41 (Actuate Therapeutics Inc) is a small molecule specific GSK-3β inhibitor in clinical trials in patients with advanced malignancies which were initiated based on its activity in a broad spectrum of pre-clinical cancer models including glioblastoma, lymphomas, neuroblastoma, and pancreatic cancers^18–22^.

While some studies have reported that inhibition of GSK-3β promotes myofibroblast differentiation and increased expression of mesenchymal markers, others show that GSK-3β inhibition blocks fibroblast activation and reduces indices of lung injury^5,13,16,23–28^. Further, our laboratory has shown that 9-ING-41 reduced myofibroblast differentiation of pleural mesothelial cells in our empyema model and improved lung function and pleural injury outcomes^23^. Here we provide evidence that GSK-3β regulates differentiation of lung myofibroblasts, that inhibition of the process can be achieved by 9-ING-41 and that administration of this inhibitor is well-tolerated and effectively blocks PF in two murine preclinical PF models.

## Materials and Methods

### Models of PF

All experiments involving animals were approved by the Institutional Animal Care and Use Committee at the University of Texas Health Science Center at Tyler. All experiments relating to animals were performed in accordance with relevant guidelines and regulations.

#### Bleomycin-induced PF in mice

PF was induced by intratracheal administration of bleomycin sulfate (Teva), as previously reported^29^ with some modifications. Briefly, C57BL/6 mice (10–12 weeks of age, ≈20 g, Jackson Laboratory, Bar Harbor ME) were first lightly anesthetized with ketamine/xylazine. Anesthetized mice were next intubated using a 20 G canula. Bleomycin (0.8U/kg in 40 µl 0.9% saline) was then administered dropwise into the canula until the entire volume had been administered. Mice were then maintained for up to 28d. For GSK-3β inhibition studies, 9-ING-41 (30 mg/kg) or vehicle control (DMSO) was administered daily by IP injection. Treatment began 14d after bleomycin administration and continued for the next 14d until the completion of the time course 28d after initiation of bleomycin-induced PF. At the conclusion of the time course, mice were assessed with pulmonary function testing and CT imaging analyses as previously reported^12,23,30,31^.

#### TGF-β-induced PF in mice

PF was initiated by intratracheal instillation of constitutively active TGF-β adenoviral vectors (Ad-TGF-β) bearing C223S/C225S mutations, as previously reported^32^ with minor modifications. Briefly, 3 × 10^8^ pfu of Ad-TGF-β or eGFP adenovirus control vector (Ad-eGFP) were administered intratracheally in a volume of 40 µl. Mice were then monitored daily until the completion of the 14d time-course. For GSK-3β inhibition studies, mice received daily 9-ING-41 or vehicle treatment as described in the previous section. 9-ING-41 was given IP 7d after administration of the adenoviral vectors. At the conclusion of the 14d time-course, pulmonary function testing and CT imaging studies were performed as previously described^12,23,30,31^.

### Lung histology, immunostaining, confocal, bright field microscopy and morphometry

Lung tissue sections from normal and IPF patients harvested at the time of transplantation were provided through a Material Transfer Agreement with the University of Michigan (Ann Arbor, Michigan). Five normal and IPF patient sections were analyzed. All tissue sections were first deparaffinized and subjected to antigen retrieval using a citrate buffer at 95 °C for 20 minutes. Tissue histology, collagen deposition and localization were initially assessed by Trichrome staining as previously described^33,34^. Immunofluorescence was used to visualize GSK-3β (Cell Signaling) in the lung tissues of bleomycin treated mice and lung fibroblasts cultured on glass coverslips^23^. Differential interference contrast (DIC) and fluorescence images were obtained using a Leica TSC SP8 confocal laser scanning microscopy system (Leica Microsystems, Inc., Heidelberg, Germany). A series of optical sections were collected at 1 μm intervals in Z-axis (17 μm). These multiple Z-series sections were then projected onto one plane at 25X optical zoom as previously described^30,34,35^. Nuclear GSK-3β expression was quantified using ImageJ as previously reported^29^.

### Fibroblast culture conditions and treatment

Normal primary human lung fibroblasts (NF) and primary human lung fibroblasts cultured from lungs harvested at the time of transplantation from patients with IPF were provided through a Material Transfer Agreement with the University of Michigan (Ann Arbor, Michigan). These cells were maintained in DMEM (Corning, Carlsbad CA) containing 10% fetal bovine serum (Life Technologies), 2% antibiotic-antimycotic (Life Technologies) and GlutaMAX (Life Technologies) as previously reported^23,29,30,34–37^. All cells were used with passage number of less than 10. Four normal and four IPF lines were used to assess responses to selected stimuli and for group comparisons.

### qPCR Analyses

Serum-starved NF and IPF fibroblasts were treated with TGF-β (5 ng/ml, R&D Minneapolis MN), thrombin (7 nM, Enzyme Research Laboratory, South Bend, IN), Factor Xa (Xa, 7 nM, Enzyme Research Laboratory), urokinase plasminogen activator (uPA, 20 nM, Sekisui Lexington MA) and plasmin (7 nM, Molecular innovations, Novi MI) for 24 h. The selected stimuli have all been implicated in the pathogenesis of PF and lung remodeling^38^. Total RNA was isolated from treated cells and transcribed into cDNA as previously described^12,23,30,31,39^. Collagen (Col) -1 and α-SMA gene expression were then determined by qPCR analyses on a Bio-Rad CFX Touch. GAPDH or GUSB was used as the loading control. All primers were purchased from Bio-Rad.

### Western blotting

Serum starved NF and IPF cells were treated with TGF-β, thrombin, factor Xa, plasmin and uPA for 48 h and prepared for Western blotting as previously reported^12,23,30^. Cell lysates and conditioned media were then collected and resolved via SDS-PAGE. Cell lysates were Western blotted for α-SMA (MAB1420, R&D), total GSK-3β (12456, Cell Signaling) and/or phosphorylated tyrosine 216 GSK-3β (Santa Cruz Biotechnology) as previously described^23,34,39^. β-actin (A1978, Sigma-Aldrich) was used as the loading control. Conditioned media was probed for secreted collagen 1 protein (Col-1, 1310-08, Southern Biotech).

### GSK-3β inhibition studies with 9-ING-41

For blockade studies, fibroblasts were treated with 9-ING-41 (10 - 0.5 µm) for 18 hours in serum-free conditions. The cells were then treated with PBS vehicle or TGF-β for 24 (RNA) or 48 h (protein). For reversal studies, serum-starved cells were first treated with TGF-β for 24 h. 9-ING-41 was then added to the cells in the presence of TGF-β. RNA was isolated after 24 h incubation, while proteins were isolated after 48 h. To confirm the role of GSK-3β in fibroblast to myofibroblast transition, the tool compound GSK-3 inhibitor TDZD-8 (40-5 µM) was also used in blockade analyses.

### Apoptosis staining

Tissue sections from the bleomycin and TGF-β models of pulmonary fibrosis were stained for apoptosis using the ApopTag® Fluorescein *In Situ* Apoptosis Detection Kit according the manufacturer’s directions. This kit recognizes and labels nicks in the DNA due to apoptosis.

### Statistics

All statistics were performed using the Mann Whitney U test or Student t-test using GraphPad Prism 8. A p-value of less than 0.05 was considered significant.

## Results

### Pulmonary GSK-3β expression is increased after TGF-β and bleomycin-induced PF

**Figure 1:**
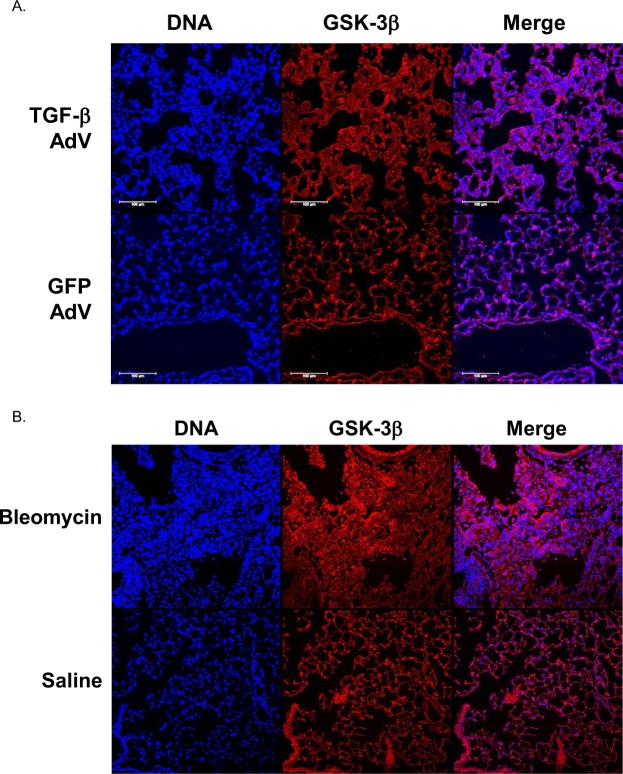
Lung tissue sections from TGF-β and bleomycin injured mice were stained for GSK-3β (red) and nuclei (blue) and imaged by confocal microscopy. GSK-3β expression was increased in TGF-β (**A**) and bleomycin-injured (**B**) mice compared to controls. Images are representative of 30 fields/slide and n = 4–6 samples/condition. Images were taken at 25X optical zoom. Bar indicates 100 µm.

To further explore the role of GSK-3β in PF, we sought to determine if expression of GSK-3β is increased in the lung tissues after induction of fibrotic pulmonary injury. To initiate these analyses, we first visualized GSK-3β expression in the lungs of mice with TGF-β and bleomycin- induced PF. Saline treated mice demonstrated ubiquitously distributed low-level expression of GSK-3β throughout the lung. Conversely, GSK-3β was upregulated within the fibrotic lesions of TGF-β- (Fig. 1A) treated mice compared to GFP adenoviral treated controls. Similar results were observed in the tissues of bleomycin treated mice compared to saline treated controls (Fig. 1B). These findings support our hypothesis that enhanced GSK-3β expression and/or activity contributes to disease progression. Total GSK-3β expression was comparable in the GFP and TGF-β adenoviral treated mice. Normal and IPF lung tissue sections also showed comparable levels of total GSK-3β (data not shown).

### GSK-3β is activated in fibroblast derived myofibroblasts

**Figure 2:**
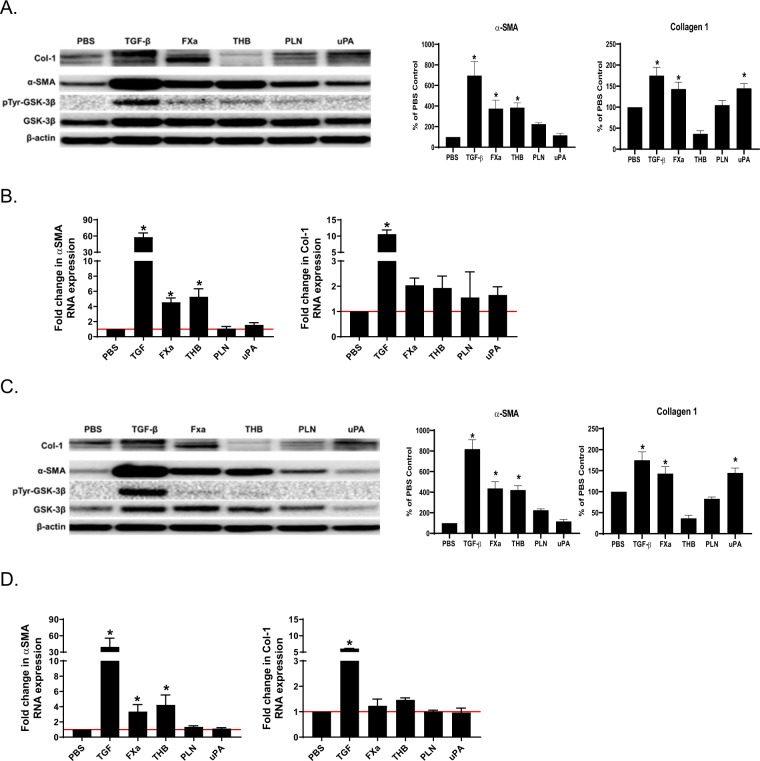
Mediators implicated in pulmonary organization induce myofibroblast differentiation of normal and IPF fibroblasts. Serum starved human fibroblasts were treated with various mediators to induce myofibroblast differentiation (TGF-β, FXa, thrombin (THB), plasmin (PLN) and uPA; see Materials and Methods). Cell lysates and conditioned medias, collected after 48 h, were then resolved by SDS-PAGE and western blotted for α-SMA, total GSK-3β, tyrosine 216 phosphorylated GSK-3β (pTyr-GSK-3β) and collagen 1 (Col-1), in NF (**A**) and IPF cells (**C**). β-actin was the loading control. α-SMA and collagen 1 expression were then quantified by densitometric analyses. Plotted data are the mean ± SEM of n = 3 independent experiments. Collagen was most prominently induced by TGF-β and FXa. Images are representative of three independent experiments. NF (**B**) and IPF (**D**) cells were treated PBS, TGF-β, Xa, thrombin, plasmin and uPA for 24 h incubation. RNA was then collected, and qPCR analyses were then performed for α-SMA and collagen 1 expression. GAPDH was the loading control. Plotted data are the mean ± SEM of n = 3–4 independent experiments.

**Figure 3:**
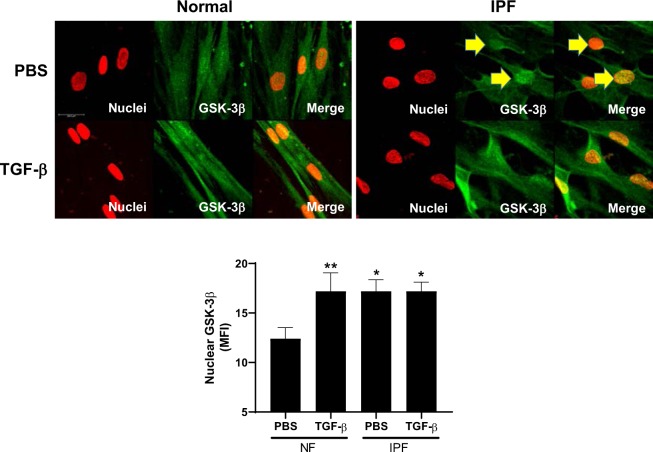
IPF fibroblasts demonstrate increased GSK-3β nuclear localization. Normal and IPF fibroblasts were seeded on glass coverslips. Serum-starved cells were then treated with TGF-β for 48 h. Cells were then fixed, permeabilized and immunostained for GSK-3β. GSK-3β (green) and nuclei (red) were then visualized by confocal microscopy. Images are representative of 10 fields/slide and n = 3 samples/condition. Images were taken at 40X optical zoom. The Mean Fluorescence Intensity (MFI) of nuclear GSK-3β was graphed as mean ± SEM. *Denotes p < 0.05, **p < 0.01. Bar indicates 25 um.

Because of the enhanced expression of GSK-3β in the lungs of mice with induced PF, we next determined the activity of GSK-3β in fibroblast-myofibroblast differentiation. Normal and IPF fibroblasts were treated with TGF-β, Factor Xa, thrombin, plasmin and uPA, mediators previously shown to induce myofibroblast transition in other cell types^34^. As anticipated, TGF-β robustly induced α-SMA expression in both normal (Fig. 2A) and IPF cells (Fig. 2C). Xa and thrombin, likewise, significantly induced α-SMA expression in both cell types. Conversely, only TGF-β and FXa significantly increased collagen 1 expression. GSK-3β expression was also enhanced in TGF-β, Xa, thrombin and plasmin treated cells. Phosphorylation of the GSK-3β activating tyrosine 216 motif was comparably enhanced by TGF-β in both NF and IPF cells. While uPA induced collagen expression in normal and IPF fibroblasts, induction of α-SMA was minimal. qPCR analyses showed significant increases in α-SMA by treatment with TGF-β, Xa and thrombin (Fig. 3A,D). TGF-β alone significantly increased Col-1 mRNA (p < 0.05).

In our recent report, we found that activated GSK-3β localizes to the nucleus in myofibroblasts^23^. To determine if GSK-3β is similarly activated in NF and IPF cells during fibroblast to myofibroblast transition, we visualized GSK-3β in normal and IPF fibroblasts in the presence and absence of TGF-β. Similar to our previous work, TGF-β treatment significantly increased GSK-3β localization to the nucleus in normal fibroblasts (Fig. 3, p < 0.01). IPF fibroblasts demonstrated significantly increased nuclear GSK-3β localization under control conditions, when compared to similarly treated NF cells. TGF-β treatment did not potentiate this affect in IPF cells. These findings suggest that GSK-3β activity is increased in IPF fibroblasts.

### 9-ING-41 blocks and reverses fibroblast-myofibroblast differentiation

**Figure 4:**
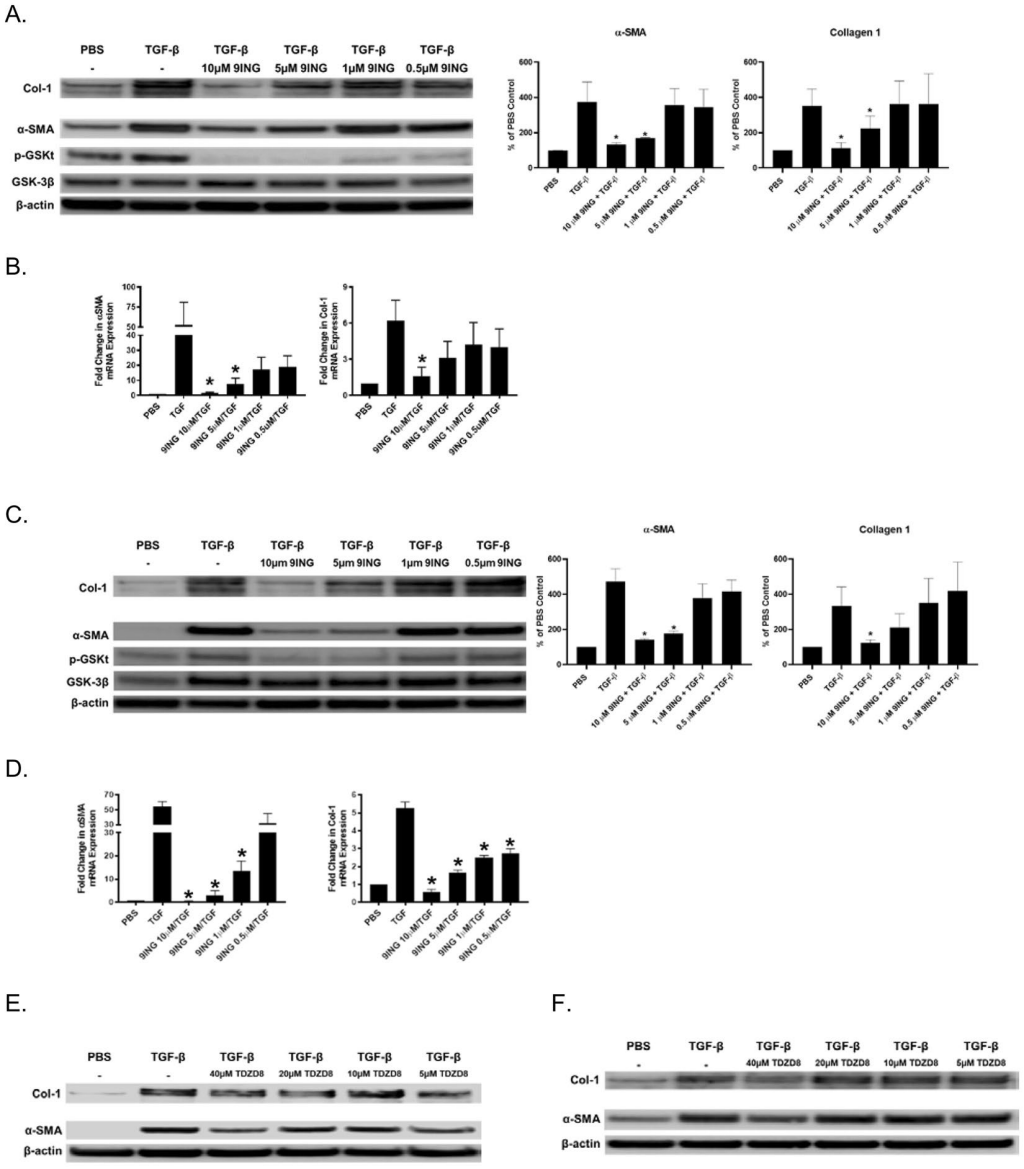
9-ING-41 blocks TGF-β mediated fibroblast to myofibroblast transition. Fibroblasts (normal, **A**,**B** and IPF, **C**,**D**) were treated with various doses of 9-ING-41 (10-0.5 µM) in serum free media. Cells were then treated with TGF-β for 48 h. Conditioned medias and cell lysates were then resolved by SDS-PAGE and immunoblotted for collagen (Col-1), α-SMA, GSK-3β, and tyrosine-216 phosphorylated GSK-3β. Under control conditions, TGF-β induced Col-1 and α-SMA protein in NF and IPF fibroblasts (**A** and **C**). 9-ING-41 (10 and 5 µM) significantly blocked TGF-β mediated induction of α-SMA and Col-1 in NF and IPF cells. Tyr-216 phosphorylation of GSK-3β (pGSKt) was likewise reduced by pretreatment with 9-ING-41 in both NF and IPF fibroblasts. Graphed data are the means of n = 3 independent experiments. Images are representative of 3–4 independent experiments. Total RNA was isolated from TGF-β treated cells in the presence or absence varying doses 9-ING-41 (10–0.5 µM). Changes in α-SMA and collagen 1 expression were then determined by qPCR analyses (**B** and **D**). GAPDH was used as the reference gene. Data are expressed as mean ± SEM. n = 3 independent experiments. *Denotes p < 0.05 compared to TGF-β control. Normal (**E**) and IPF (**F**) fibroblasts were treated with varying doses of TDZD-8 (40-5 µM) prior to the addition of TGF-β. Cell lysates and conditioned media were then resolved via SDS-PAGE and probed for changes α-SMA and collagen. β-actin was the loading control. TDZD-8 modestly reduced α-SMA and collagen induction by TGF-β at the highest dose (40 µM). Images are representative of two independent experiments.

Because GSK-3β was activated in TGF-β treated NF and IPF fibroblasts, we next determined the effect of 9-ING-41 on fibroblast to myofibroblast differentiation. In blockade studies, normal and IPF fibroblasts were treated with TGF-β in the presence and absence of decreasing concentrations of the 9-ING-41 (10–0.5 µM). The two highest doses of 9-ING-41 (10 and 5 µM) significantly blocked induction of α-SMA and collagen-1 in both normal and IPF cells (Fig. 4A,C). While TGF-β treatment increased Tyr-216 phosphorylation of GSK-3β, this phosphorylation event was reduced in 9-ING-41 treated cells. 9-ING-41 also blocked PI3K/AKT signaling (data not shown). Similar results were found in parallel qPCR analyses (Fig. 4B,D). These results were confirmed with a mechanistically different, GSK-3β inhibitor, TDZD-8. Although TDZD-8 attenuated TGF-β mediated induction myofibroblast biomarkers (α-SMA and collagen), only the highest dose (40 µM) was effective at blocking TGF-β mediated increases in α-SMA and collagen (Fig. 4E,F) in both NF and IPF cells. These analyses showed that GSK-3β inhibition blocks fibroblast to myofibroblast transition.

**Figure 5:**
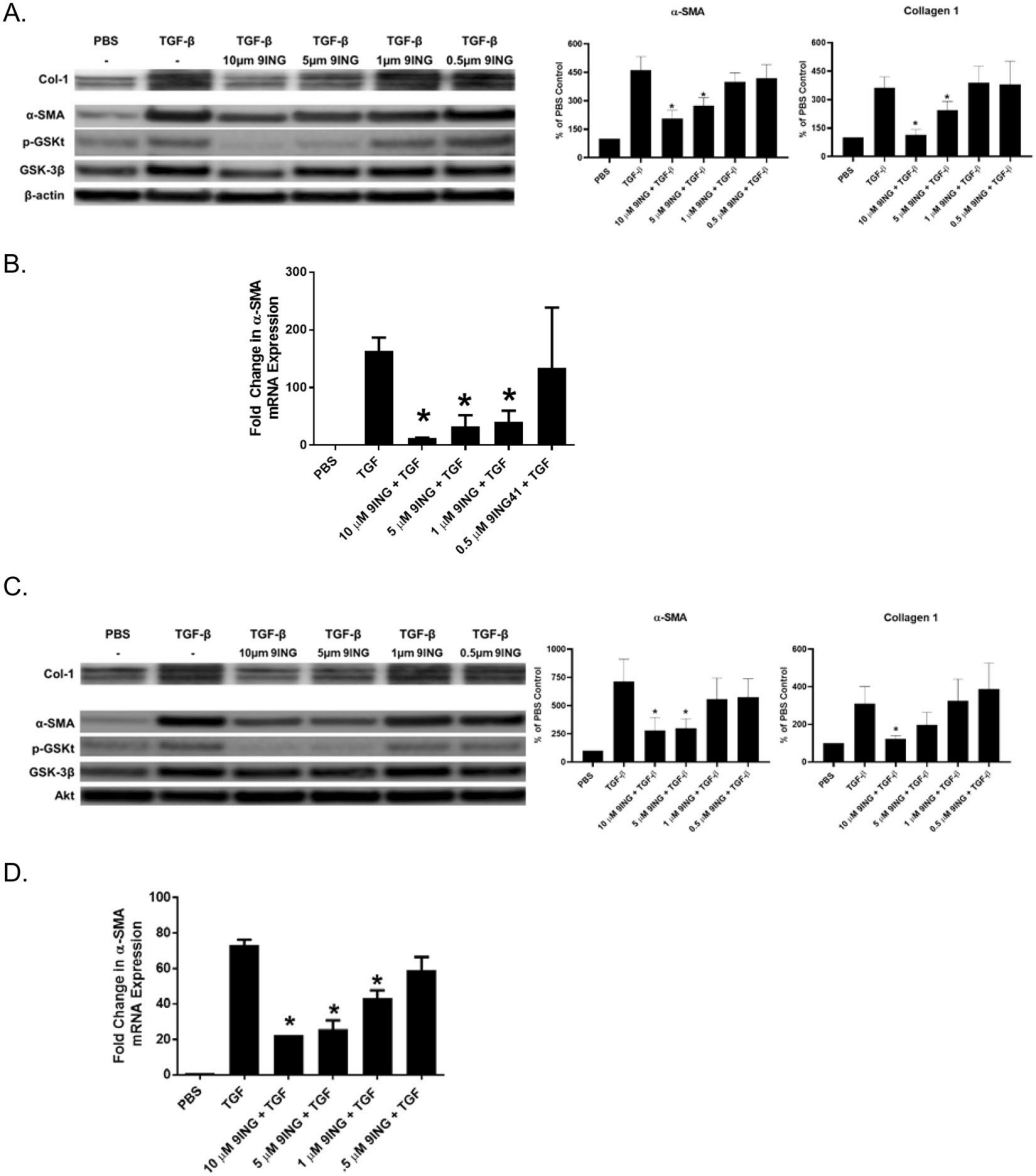
9-ING-41 reverses established fibroblast to myofibroblast transition. Serum-starved NF and IPF cells were treated with TGF-β for 24 hours. Varying doses of 9-ING-41 (10-5 µM) were then added to the TGF-β–treated cells and allowed to incubate for 48 hours. Conditioned media and lysates from NF (**A**) and IPF (**C**) were resolved by SDS-PAGE and immunoblotted for collagen (Col)-1 and α-smooth muscle actin (α-SMA). Lysates were also immunoblotted for GSK-3β phosphorylation at tyrosine 216. β-Actin was used as loading control. For quantitative PCR (qPCR) analyses varying doses of 9-ING-41 (10 to 0.5 µM) were added to TGF-β–treated normal (**B**) and IPF (**D**) cells and then allowed to incubate for 24 hours. Total RNA was then isolated and transcribed into cDNA. α-SMA expression was determined by qPCR analyses. GAPDH served as the reference gene. Data are expressed as means ± SEM. n = 3 independent experiments. ∗p < 0.05 versus TGF-β treatment.

We next determined if 9-ING-41 could reverse established myofibroblast differentiation (Fig. 5). For these experiments, cells were treated with TGF-β for 24 hours prior to the addition of 9-ING-41 (10–0.5 µM) for 24 hours. Similar to the blockade studies described above, the two highest doses of 9-ING-41 (10 and 5 µM) significantly reversed induction of the myofibroblast phenotype. Specifically, TGF-β mediated induction of α-SMA and collagen 1 expression was reversed in 9-ING-41 treated NF (Fig. 5A) and IPF (Fig. 5C) cells. Similar results were seen in qPCR analyses (Fig. 5B,D). GSK-3β phosphorylation at Tyr-216 was likewise reduced by treatment with 9-ING-41 in NF (Fig. 5A) and IPF (Fig. 5C) cells.

### Therapeutic targeting of GSK-3β with9-ING-41 improves PF *in vivo*

**Figure 6:**
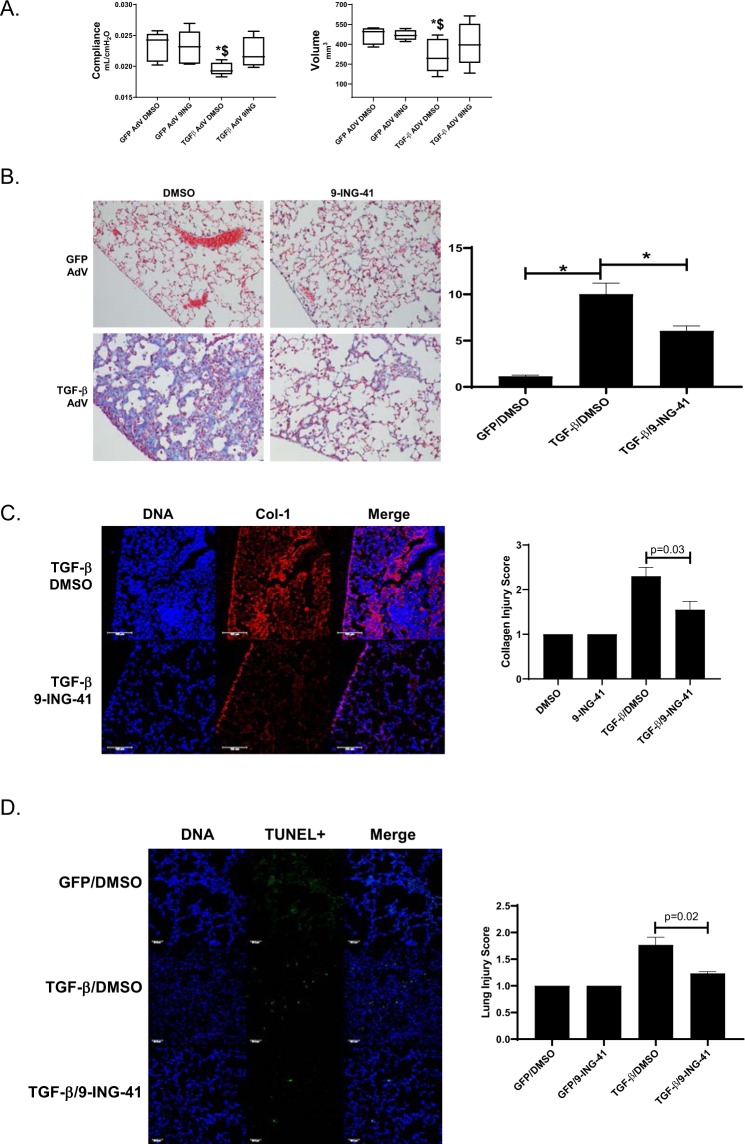
9-ING-41-treated mice demonstrate reduced TGF-β mediated PF. Mice were intratracheally administered TGF-β adenovirus to induce pulmonary fibrosis. After 7d, mice received daily intraperitoneal injections of 9-ING-41 (30 mg/kg) for the next 7d. At the completion of the 14d time course, lung compliance and volumes were determined. A, Significant decrements in lung compliance and volume were reversed by 9-ING-41 treatment. Data are expressed as a mean ± SEM. n = 6 mice/condition. *Indicates a p < 0.05 compared to GFP adenovirus DMSO treatment. ^$^Denotes p < 0.05 compared to GFP adenovirus 9-ING-41 treatment. B. Lung tissue sections from DMSO and 9-ING-41 treated mice were Trichrome stained to show areas of injury and collagen (blue). Images were taken at 20X optical zoom. Images are representative of 30 fields/slide/condition. n = 6–7 mice. Lung injury score data are expressed as means ± SEM. (**C**) Lung tissue sections from TGF-β adenoviral mice treated with vehicle and 9-ING-41 mice were immunostained to visualize collagen (Col-1) deposition (red) and nuclei (blue) by confocal microscopy. Images were taken at 25x optical zoom. Collagen injury score data are expressed as mean ± SEM. n = 6 mice/condition. Bar indicates 100 µm. (**D**) Lung tissue sections from GFP and TGF-β adenovirus infected mice were stained for apoptosis by fluorescent TUNEL stain (green) and nuclei (blue). Images were taken at 20X optical zoom. Images are representative of 15 fields/slide/condition and n = 5–6 samples/condition. Data are expressed as mean ± SEM. p denotes a p < 0.05. Bar indicates 50 µm.

To further interrogate the role of GSK-3β in PF, we first induced PF using TGF-β adenovirus (Fig. 6). In these studies, 9-ING-41 treatment was initiated 7 days after instillation of the adenoviral vectors. 9-ING-41 significantly improved decrements (p < 0.05) in lung compliance and lung volume compared to TGF-β adenoviral treated mice (Fig. 6A). Lung injury by morphometry was next analyzed using a 3-point scale, where 1 = no scarring and 3 = extensive scarring. 9-ING-41 treatment significantly reduced the lung injury score of TGF-β adenovirus induced PF in mice (Fig. 6B, p = 0.02). Collagen deposition (Fig. 6C) was likewise significantly reduced by 9-ING-41 treatment (Fig. 6C, p = 0.03). TGF-β adenovirus treated mice also demonstrated significantly increased numbers of apoptotic cells compared to GFP adenovirus controls. 9-ING-41 treatment significantly reduced the number of apoptotic cells^40,41^, presumably including alveolar epithelial cells, in TGF-β treated mice compared to GFP adenoviral controls (Fig. 6D).

**Figure 7:**
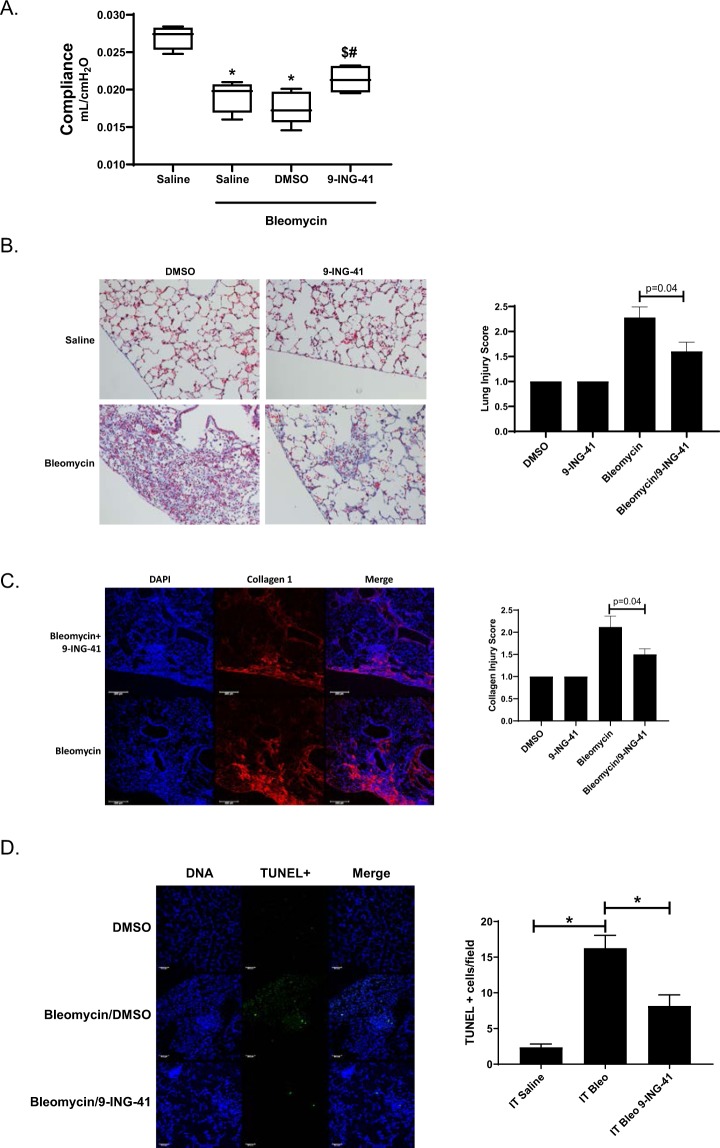
9-ING-41-treated mice demonstrate reduced bleomycin mediated PF. (**A**) Anesthetized C57Bl/6 J mice were intratracheally administered 0.8U bleomycin/kg. After 14d mice were administered vehicle control (DMSO) or 9-ING-41 (30 mg/kg) by daily intraperitoneal injection for the next 14d. At the completion of the 28d time-course lung compliance and volumes were determined with the Scireq flexivent and by chest CT imaging, respectively. 9-ING-41 treated mice demonstrated significantly better lung compliance and lung volumes than DMSO-treated controls. *Indicates a p < 0.05 compared to saline control. ^$^Denotes p < 0.05 compared to bleomycin/saline treated mice. ^#^Denotes a p < 0.05 compared to bleomycin/DMSO treated mice. (**B**) Lung tissue sections from DMSO and 9-ING-41 treated mice were Trichrome stained to show areas of injury and collagen (blue). Images were taken at 20X optical zoom. Images are representative of 30 fields/slide/condition and n = 6 mice. Lung injury score data are expressed as means ± SEM. (**C**) Lung tissue sections from vehicle and 9-ING-41 treated mice were immunostained to visualize collagen deposition (red) by confocal microscopy. Images were taken at 10x optical zoom. Bar indicates 100 µM. (**D**) Lung tissue sections from saline, bleomycin and bleomycin/9-ING-41 treated mice were stained for apoptosis by fluorescent TUNEL stain (green). Images were taken at 20X optical zoom. Images are representative of 15 fields/slide/condition. Data are expressed as mean ± SEM. p denotes a p < 0.05. n = 5–6 samples/condition. Bar indicates 50 µm.

We next induced PF using the intratracheal bleomycin model. In these experiments, mice were injured 14 days prior to initiating treatment with DMSO vehicle or GSK-3β inhibitor; 9-ING-41. Bleomycin injured mice were then treated with 9-ING-41 or DMSO vehicle control for 14 days (Fig. 7). At 28 days post injury, both untreated and vehicle-treated mice demonstrated significant reductions in lung compliance compared to uninjured mice (Fig. 7A). However, 9-ING-41 treated mice showed significantly improved lung compliance compared to saline and vehicle-treated controls (p < 0.05).

Lung tissue sections from DMSO and 9-ING-41 treated mice were next trichrome-stained to show changes in lung architecture and collagen/neomatrix deposition. While DMSO treated mice showed pronounced injury and areas of intense collagen deposition, 9-ING-41 treated mice had significantly fewer areas of injury and demonstrated less collagen deposition (Fig. 7B). These findings were confirmed by immunological staining for collagen 1 and imaging by confocal microscopy that showed marked collagen deposition in DMSO treated mice (Fig. 7C). Conversely, 9-ING-41 treatment significantly reduced areas of intense collagen deposition (p = 0.04). Because apoptosis of epithelial cells is strongly implicated as a driver of PF in the bleomycin model and other forms of PF^29^, we next analyzed apoptosis in bleomycin mediated PF in the presence and absence of 9-ING-41 (Fig. 7D, p < 0.05). Bleomycin treatment significantly increased the number of TUNEL positive cells lining and within the alveolar walls. Conversely, 9-ING-41 treatment significantly reduced the number of apoptotic cells^40,41^ in bleomycin-treated mice compared to vehicle treated controls (p < 0.05). These findings strongly support our hypothesis that the therapeutic targeting of GSK-3β with the novel inhibitor, 9-ING-41, reduces myofibroblast differentiation, collagen deposition, and subsequent PF *in vivo*.

## Discussion

The aberrant accumulation of matrix-producing myofibroblasts in fibrotic foci and areas of remodeling characterizes interstitial lung diseases, including IPF. Fibroblast to myofibroblast differentiation is believed to significantly contribute to this process. Although GSK-3β had been shown to be important in the treatment of some cancers and the pathogenesis of fibrosis in other organs^13–16,23,24,42,43^, its role in myofibroblast differentiation and the progression of PF has remained unclear until the present time. In this study, we show that GSK-3β activation is critical for fibroblast to myofibroblast differentiation. Further, a novel clinically relevant GSK-3β inhibitor; 9-ING-41, now demonstrating promise in human neoplasia^18–20^ effectively reversed lung injury in two distinct models of PF.

The role of GSK-3β in fibroblast to myofibroblast transition has been controversial. Studies by Baarsma and colleagues show that GSK-3 inhibition attenuates induction of α-SMA and fibronectin expression. Further, they show that this mechanism is dependent on GSK-3-mediated activation of CREB signaling^13^. These studies however were limited to analyses in a fibroblast cell line. Similar studies by Liu *et al*. showed that GSK-3 inhibition protected against PF by increasing cellular autophagy^26^. Conversely, studies by Xia *et al*. found that GSK-3β is inactivated in IPF-derived fibroblasts and that subsequent aberrant activation of β-catenin may contribute to cellular differentiation. Similar results were found in peritoneal mesothelial cells, in which activation of GSK-3β protected against MesoMT^42–44^ while promoting cell death^45^. These disparate findings in the aggregate support our hypothesis that GSK-3β promotes PF and offer a strong rationale to investigate its role in this condition, as we did in this study.

Our data here clearly show that inhibition of GSK-3β improves physiologic outcomes in two models of PF and that the effects are associated with inhibition of fibroblast to myofibroblast transition and subsequent matrix deposition. To our knowledge, this represents the first evidence along these lines and suggests that this approach merits further consideration for further translational investigation. We first showed that GSK-3β expression was enhanced in the lungs of mice with bleomycin induced PF. Similar changes were not observed in tissue sections of IPF patients or in the TGF-β PF model. We posit the chronicity of the disease in IPF patients may have muted the changes in GSK-3β expression. The variable GSK-3β expression we observed in our subacute models likely reflects the different agents we used to induce PF, possible differences in the durability or intensity of the response, or differences in the contributions of levels of GSK-3β to the different forms of PF. We next showed that diverse mediators could induce myofibroblast differentiation of NF and IPF cells. TGF-β, however, was the most potent inducer of α-SMA and collagen in these cells.

Although GSK-3β is a constitutively active kinase, its activity can be increased by phosphorylation of Tyr-216 and localization to the nucleus and^46,47^. In our previous work, we found that a diverse group of mediators (TGF-β, FXa, thrombin, plasmin and uPA) potently induced tyr-216 GSK-3β phosphorylation in HPMCs. In both NF and IPF cells, we found that TGF-β enhanced GSK-3β Tyr-216 phosphorylation to a much greater extent than any of the other tested mediators. We also found that GSK-3β localized primarily to the nucleus of NF and IPF. Further, TGF-β enhancement of GSK-3β localization to the nucleus was limited to the IPF cells. Because we previously showed that GSK-3β was localized primarily to the cytoplasm of unstimulated PMCs, we now hypothesize that the role of GSK-3β in myofibroblast differentiation may vary by cell type. These findings will be studied in greater detail in subsequent studies. They further support our hypothesis that GSK-3β phosphorylation at Tyr-216 and localization to the nucleus are drivers of myofibroblast differentiation.

To determine the role of GSK-3β in fibroblast to myofibroblast transition, we initially attempted to directly reduce its expression with siRNA- and GSK-3β shRNA-encoding adenoviral vectors. However, we were unable to sufficiently reduce GSK-3β expression for subsequent analyses. As such, our studies focused on the use of GSK-3β-targeting pharmacologic inhibitors to assess the contribution of GSK-3β in fibroblast to myofibroblast transition. For these studies, we used the highly efficacious GSK-3β inhibitor; 9-ING-41. We previously showed that 9-ING-41 strongly blocked and reversed MesoMT and reduced Tyr-216 phosphorylation^23^. Further, 9-ING-41 effectively blocked MesoMT at lower concentrations than TDZD-8. In the studies presented here, we found similar results, as 9-ING-41 treatment blocked TGF-β-mediated myofibroblast differentiation at doses 8 times less than TDZD-8. While the same concentration of 9-ING-41 (10 µM), was effective for pleural mesothelial cells, higher concentrations of TDZD-8 were required to effectively block lung fibroblast to myofibroblast differentiation (40 µM versus 20 µm). This difference is most likely a consequence of the increased efficacy and half-life of 9-ING-41 compared to other GSK-3β inhibitors^48^.

We next confirmed that 9-ING-41 could reverse established myofibroblast differentiation. TGF-β can induce detectable markers (α-SMA and collagen) of fibroblast to myofibroblast transition in as little as 24 h^29,34,39^. We found that induction of these biomarkers was reversed by treatment with 9-ING-41. Western blotting and qPCR analyses showed that the highest two concentrations of 9-ING-41 effectively reversed α-SMA and collagen 1 induction. Further, GSK-3β phosphorylation was also markedly reduced. These findings strongly support our hypothesis that GSK-3β inhibition by reducing Tyr-216 phosphorylation represents a therapeutic target for the treatment of PF, providing the rationale for *in vivo* analyses.

Because of our prior experience with 9-ING-41 in mice^23^, we chose to use a daily 30 mg/kg dose of 9-ING-41. In this study, 9-ING-41-treated mice tolerated this dose and showed no overt signs of toxicity. 9-ING-41 significantly improved lung function in both models of PF. In the bleomycin study, 9-ING-41 treatment significantly improved lung function and lung volumes compared to vehicle treated mice. 9-ING-41 treatment did not, however, restore lung function to control levels. While collagen deposition was reduced by 9-ING-41 treatment, residual areas of lung injury and hyperplasia were observed. These findings suggest that 9-ING-41 treatment prevented scarring resulting from fibroblast to myofibroblast transition but was not fully protective at the doses we used. Further, the fact that 9-ING-41 reduced overall lung and, presumably, epithelial apoptosis in both the TGF-β and bleomycin models strongly suggests that preservation of epithelial cell viability contributes to protection of the lung architecture. Additional studies with longer treatment courses may show continued improvement compared to vehicle control with values approaching normal levels.

In summary, this report provides evidence that GSK-3β plays a critical role in myofibroblast differentiation and subsequent PF. While numerous factors may contribute to disease progression, TGF-β likely plays a central role in this process. As TGF-β is locally expressed and implicated in the pathogenesis of PF^49,50^, we now demonstrate that it too can activate GSK-3β during induction of fibroblast to myofibroblast transition. We also found that other mediators of neomatrix remodeling can drive myofibroblast differentiation. As in other cell types^25,31,34,39,51^, these factors may likewise activate GSK-3β *in vivo*, thereby promoting PF. While inhibition of GSK-3β with 9-ING-41 can attenuate myofibroblast differentiation *in vitro*, our findings show that pharmacologic targeting of GSK-3β *in vivo* is effective and generally well tolerated. Our findings also provide a strong rationale for advancement of 9-ING-41 as a candidate new therapeutic for the treatment of patients with PF.

## Data Availability

No datasets were generated or analyzed during the current study.
